# Supplementary material for: Multi‐dataset identification of innovative feature genes and molecular mechanisms in keratoconus
Source: J Cell Mol Med. 2024 Sep 19;28(18):e70079. doi: 10.1111/jcmm.70079 (PMC11412914; doi:10.1111/jcmm.70079)
Supplement: Supplementary file 2 — Material S2. [file JCMM-28-e70079-s002.zip › 7.GSEA analysis/5.RNF217-AS1/KEGG_PROTEIN_EXPORT.html]

Details for gene set KEGG\_PROTEIN\_EXPORT[GSEA]

|  || Dataset | RNF217-AS1.group.cls#h\_versus\_l |
| Phenotype | group.cls#h\_versus\_l |
| Upregulated in class | h |
| GeneSet | KEGG\_PROTEIN\_EXPORT |
| Enrichment Score (ES) | 0.25889358 |
| Normalized Enrichment Score (NES) | 0.83177197 |
| Nominal p-value | 0.74174756 |
| FDR q-value | 1.0 |
| FWER p-Value | 1.0 |
Table: GSEA Results Summary

  

Fig 1: Enrichment plot: KEGG\_PROTEIN\_EXPORT      
 Profile of the Running ES Score & Positions of GeneSet Members on the Rank Ordered List

  

| SYMBOL | TITLE | RANK IN GENE LIST | RANK METRIC SCORE | RUNNING ES | CORE ENRICHMENT || 1 | SEC61A2 | na | 1167 | 0.422 | 0.2589 | Yes |
| 2 | SRP19 | na | 10706 | 0.074 | 0.0357 | No |
| 3 | IMMP2L | na | 12157 | 0.053 | 0.0309 | No |
| 4 | SRP54 | na | 12366 | 0.050 | 0.0599 | No |
| 5 | SEC61G | na | 15718 | 0.011 | -0.0287 | No |
| 6 | IMMP1L | na | 16404 | 0.005 | -0.0452 | No |
| 7 | SRP9 | na | 16640 | 0.002 | -0.0504 | No |
| 8 | SEC61B | na | 16722 | 0.001 | -0.0517 | No |
| 9 | SRP68 | na | 16774 | 0.001 | -0.0526 | No |
| 10 | SEC11A | na | 17134 | -0.003 | -0.0609 | No |
| 11 | SRP14 | na | 18463 | -0.015 | -0.0886 | No |
| 12 | SPCS3 | na | 18997 | -0.020 | -0.0901 | No |
| 13 | SPCS1 | na | 19489 | -0.025 | -0.0868 | No |
| 14 | SPCS2 | na | 19920 | -0.029 | -0.0790 | No |
| 15 | SRP72 | na | 20340 | -0.033 | -0.0679 | No |
| 16 | SEC11C | na | 21629 | -0.047 | -0.0727 | No |
| 17 | OXA1L | na | 23723 | -0.071 | -0.0835 | No |
| 18 | SEC63 | na | 24044 | -0.076 | -0.0401 | No |
| 19 | SRPRB | na | 24571 | -0.084 | 0.0028 | No |
| 20 | SEC61A1 | na | 25161 | -0.093 | 0.0502 | No |
| 21 | HSPA5 | na | 25825 | -0.104 | 0.1032 | No |
| 22 | SEC62 | na | 30438 | -0.221 | 0.1236 | No |
Table: GSEA details [plain text format]

  

Fig 2: KEGG\_PROTEIN\_EXPORT      
 Blue-Pink O' Gram in the Space of the Analyzed GeneSet

  

Fig 3: KEGG\_PROTEIN\_EXPORT: Random ES distribution      
 Gene set null distribution of ES for **KEGG\_PROTEIN\_EXPORT**
